# Monocytes Differentiate to Immune Suppressive Precursors of Metastasis-Associated Macrophages in Mouse Models of Metastatic Breast Cancer

**DOI:** 10.3389/fimmu.2017.02004

**Published:** 2018-01-17

**Authors:** Takanori Kitamura, Dahlia Doughty-Shenton, Luca Cassetta, Stamatina Fragkogianni, Demi Brownlie, Yu Kato, Neil Carragher, Jeffrey W. Pollard

**Affiliations:** ^1^MRC Centre for Reproductive Health, Queen’s Medical Research Institute, The University of Edinburgh, Edinburgh, United Kingdom; ^2^Edinburgh Phenotypic Assay Centre, Queen’s Medical Research Institute, The University of Edinburgh, Edinburgh, United Kingdom; ^3^Department of Developmental and Molecular Biology, Albert Einstein College of Medicine, New York, NY, United States; ^4^Cancer Research UK Edinburgh Centre, MRC Institute of Genetics & Molecular Medicine, The University of Edinburgh, Edinburgh, United Kingdom

**Keywords:** breast cancer, metastasis, macrophage, myeloid-derived suppressor cell, immune suppression, CD8^+^ T cell

## Abstract

Metastasis-associated macrophages (MAMs) play pivotal roles in breast cancer metastasis by promoting extravasation and survival of metastasizing cancer cells. In a metastatic breast cancer mouse model, we previously reported that circulating classical monocytes (C-MOs) preferentially migrated into the tumor-challenged lung where they differentiated into MAMs. However, the fate and characteristics of C-MOs in the metastatic site has not been defined. In this study, we identified that adoptively transferred C-MOs (F4/80^low^CD11b^+^Ly6C^+^) differentiated into a distinct myeloid cell population that is characterized as F4/80^high^CD11b^high^Ly6C^high^ and gives rise to MAMs (F4/80^low^CD11b^high^Ly6C^low^) within 18 h after migration into the metastatic lung. In mouse models of breast cancer, the CD11b^high^Ly6C^high^ MAM precursor cells (MAMPCs) were commonly found in the metastatic lung, and their accumulation was increased during metastatic tumor growth. The morphology and gene expression profile of MAMPCs were distinct from C-MOs and had greater similarity to MAMs. For example MAMPCs expressed mature macrophage markers such as CD14, CD36, CD64, and CD206 at comparable levels with MAMs, suggesting that MAMPCs have committed to a macrophage lineage in the tumor microenvironment. MAMPCs also expressed higher levels of *Arg1, Hmox1*, and *Stab1* than C-MOs to a comparable level to MAMs. Expression of these MAM-associated genes in MAMPCs was reduced by genetic deletion of colony-stimulating factor 1 receptor (CSF1R). On the other hand, transient CSF1R blockade did not reduce the number of MAMPCs in the metastatic site, suggesting that CSF1 signaling is active in MAMPCs but is not required for their accumulation. Functionally MAMPCs suppressed the cytotoxicity of activated CD8^+^ T cells *in vitro* in part through superoxide production. Overall, our results indicate that immediately following migration into the metastatic tumors C-MOs differentiate into immunosuppressive cells that have characteristics of monocytic myeloid-derived suppressor cell phenotype and might be targeted to enhance efficacy of immunotherapy for metastatic breast cancer.

## Introduction

Breast cancer is the most common cancer in women worldwide, accounting for 23% of the total new cancer cases (1). The mortality rate of breast cancer has been decreasing due to the development of early detection techniques and improvement in treatment (1). However, breast cancer cells frequently metastasize to the bone and lung, which dramatically reduces 5-year survival of breast cancer patients to less than 25% (2). Indeed, data show that survival of metastatic breast cancer patients has not significantly improved over the past 30 years (3). This depressing statistic indicates the requirement of novel approaches that efficiently block metastatic tumor development.

Attractive targets for improvements in therapy are tumor-infiltrating immune cells such as regulatory T cells, tumor-associated neutrophils, myeloid-derived suppressor cells (MDSCs), and tumor-associated macrophages (TAMs), as these cells play pivotal roles in the establishment of metastatic tumors (4). In particular, TAMs have been shown to be critical promoters of metastatic breast cancer development following early experiments that showed marked suppression of tumor progression and metastasis by genetic macrophage depletion in a mouse model of breast cancer in which mammary tumors are caused by the mammary epithelial restricted polyoma middle T oncoprotein (PyMT) expression (5). In addition, many studies have correlated poor prognosis of breast cancer patients with high infiltration of TAMs into the tumor (6). Data from mouse models of metastatic breast cancer have also defined mechanisms of this metastasis promotion indicating that TAMs support tumor cell invasion and intravasation at the primary sites (7), enhance angiogenesis (8), transmit survival signals to the metastasizing tumor cells (9), and promote extravasation and persistent growth of tumor cells at the metastatic site (10). In addition, there is preliminary evidence that TAMs are involved in immunosuppression. For example, TAMs express high levels of programmed death ligand 1 (PD-L1), a ligand for immune-checkpoint receptor that restricts CD8^+^ T cell activities (11, 12), and the targeting of TAMs improves efficacy of the check-point inhibitors in a pancreatic ductal carcinoma model in mice (13). It is also reported that TAMs suppress CD8^+^ T cell-mediated anti-tumor immunity in the mammary tumor of PyMT mice under treatment with chemotherapy (14). Therefore, TAMs represent important potential therapeutic target to treat metastatic breast cancer (4).

One of the most extensively explored strategies to target TAMs is the inhibition of colony-stimulating factor 1 receptor (CSF1R) (12). Antagonists or blocking antibodies against CSF1R suppress the accumulation of TAMs as well as changing their phenotype, enhance CD8^+^ T cell-mediated antitumor immune responses, and prevent disease progression or primary tumor growth in mouse models of glioblastoma, pancreatic, colon, and breast cancer (13–16). These data based on genetically engineered mouse models of the primary tumor suggest that TAM intervention by CSF1R inhibition is an attractive strategy to block environmental support for malignant tumor development and to improve therapeutic efficacy of CD8^+^ T cell-based immunotherapy.

For therapeutic TAM intervention aimed at blocking the metastatic tumor expansion, a better understanding of macrophages in the metastatic sites is important since macrophages change their phenotypes in response to environmental factors and these might be different between the primary and metastatic tumors (17). In mouse models of metastatic breast cancer, there are at least two distinct macrophage populations characterized as F4/80^+^CD11b^low^CD11c^high^ and F4/80^+^CD11b^high^CD11c^low^ in the lung with metastatic tumors (10). The CD11c^high^ macrophage population consists of alveolar macrophages that also exist in the normal lung (18, 19). In contrast, the CD11b^high^ macrophages markedly accumulate in the tumor-challenged lung but are significantly less in the normal lung (10). In an experimental metastasis model using Met-1 mouse breast cancer cells on a FVB genetic background, depletion of these CD11b^high^ metastasis-associated macrophages (MAMs) but not CD11c^high^ resident macrophages (RMACs) reduces the number and size of metastatic foci (10). In this model, a subset of monocytes characterized as CD11b^+^Ly6C^+^ [classical monocytes (C-MOs)] preferentially migrates to the tumor-challenged lung *via* a chemokine receptor CCR2, and inhibition of their recruitment results in the reduction of the number of MAMs (CD11b^high^Ly6C^low^) and metastatic tumor load in the lung (20). In another experimental metastasis model using E0771-LG mouse breast cancer cells on a C57BL/6 background, adoptively transferred CD11b^+^Ly6C^+^ C-MOs differentiate to a CD11b^high^Ly6C^low^ population within 42 h posttransfer (21). Although a minor macrophage population in the normal lung called interstitial macrophages is also characterized as CD11b^+^CD11c^low^ (18, 19), these cells are not rapidly replenished by C-MOs (22) and their accumulation by bacterial CpG DNA does not require CCR2 (23). Collectively, these data indicate that the circulating C-MOs differentiate into MAMs at the metastatic sites, which promotes the establishment of metastatic tumors. Therefore, C-MOs in the differentiation process at the metastatic site can be a novel therapeutic target for the treatment of metastatic breast cancer, and thus it is important to understand their dynamics and characteristics after infiltrating the metastatic tumors.

In this article, we have identified that circulating C-MOs differentiate into a distinct myeloid cell population characterized as CD11b^high^Ly6C^high^ in the metastatic lung where they further differentiate into MAMs. The CD11b^high^Ly6C^high^ MAM precursor cells (MAMPCs) expressed mature macrophage markers, and their gene expression profile was similar with that of MAMs but distinct from C-MOs. We also found that accumulation of the CD11b^high^Ly6C^high^ cells was increased when micro-metastasis started to outgrow, and was not suppressed by blockade of CSF1R. We further identified that the MAMPCs suppressed cytotoxic ability of CD8^+^ T cells through reactive oxygen species (ROS)-mediated but checkpoint ligands-independent mechanism. These results indicate that C-MOs recruited to the metastatic tumors produce immune suppressive precursor MAMs that may not be targeted by CSF1R antagonists or checkpoint inhibitors.

## Materials and Methods

### Mice

MMTV-PyMT mice (24) on the C57BL/6 background were obtained from Dr. Sandra J. Gendler (Mayo Clinic College of Medicine) who had backcrossed PyMT mice established by Dr. William J Muller (McGill University, Montreal, Canada) originally on the FVB background. To analyze the lung with metastatic tumors, we used female PyMT mice on the C57BL/6 background at 20–25 weeks of age. *Csf1r-*EGFP (MacGreen) (25) mice on the C57BL/6 background were obtained from Dr. David Hume (University of Edinburgh). Conditional CSF1R knockout (*Csf1r* cKO) mice (i.e., rtTA:tetO-Cre:*Csf1r*^F/F^) were obtained by crossing the B6.Cg-*Csf1r*^tm1Jwp^/J (*Csf1r*^F/F^) mice (26) with ROSA-rtTA and tetO-Cre mice (Jackson lab) (21). Animals were housed and bred under standard conditions of care. All procedures involving mice were conducted in accordance with licensed permission under the UK Animal Scientific Procedures Act (1986) (Home Office license number PPL 70/8065) and Institutional Animal Care & Use Committee of the Albert Einstein College of Medicine (20120304).

### Tumor Cell Lines

Met-1 mouse mammary tumor cells derived from the MMTV-PyMT tumor in FVB mice (27) and highly metastatic derivative of E0771 mouse mammary adenocarcinoma cells derived from a medullary cancer in C57BL/6 mice (E0771-LG) (21) were cultured in DMEM supplemented with 10% v/v FBS, 100 U/mL penicillin, and 100 µg/mL streptomycin. E0771-LG cells were manipulated to express firefly luciferase (E0771-LG:Fl) or nuclear localized red fluorescent protein (mKate) (E0771-LG:NLR) to detect the cells by *in vivo* bioluminescence imaging or *in vitro* fluorescence microscopy, respectively. We have confirmed that all cells were negative for mycoplasma.

### Breast Cancer Metastasis Models in Mice

As experimental models of metastatic breast cancer, we injected 1 × 10^6^ of E0771-LG or Met-1 cells into the tail vein of C57BL/6 or FVB mice (7-week-old female), respectively. At 7–14 days (E0771-LG) or 21 days (Met-1) posttumor cell injection, we euthanized the mice and isolated the blood and lung to prepare samples for flow cytometry or H&E staining.

### *In Vivo* Bioluminescence Imaging

We intraperitoneally injected d-luciferin in PBS (GoldBio, 1.5 mg/100 μL/20 g mouse) into anesthetized E0771-LG:Fl tumor-bearing mice. Bioluminescence from the luciferase-expressing tumor cells was imaged using Photon Imager Optima (Biospace Lab), and photon counts (photon/second/cm^2^/sr) in the lung area were quantified by image analysis software (M3 Vision, Biospace Lab).

### Adoptive Transfer of Monocytes

We isolated C-MOs from the bone marrow of MacGreen mice by Monocyte Isolation Kits (Miltenyi). 5 × 10^5^ of the GFP^+^ C-MOs were transferred into C57BL/6 mice that have received intravenous injection of E0771-LG:Fl cells. Pulmonary tumor burdens in the recipient mice were determined by bioluminescence imaging 1 day before monocyte transfer (10 days after tumor injection). Eighteen, 42, 66, or 90 h after the C-MO transfer, we euthanized the animals and isolated the blood and lung for flow cytometry.

### Flow Cytometry and Sorting

Single-cell suspensions from perfused lungs were prepared *via* enzymatic digestion using the Lung Dissociation Kit (Miltenyi) and following filtration through a 40 µm cell strainer. Red blood cells in the lung digestions and blood samples were removed using RBC lysis buffer (Biolegend). The samples were blocked with antimouse CD16/CD32 antibody (BD bioscience), and stained with DAPI and fluorescent antibodies to following antigens; CD45 (30-F11), F4/80 (BM8), CD11b (M1/70), Ly6C (HK1.4), CD115 (AFS98), Ly6G (1A8), CD11c (N418), CD14 (Sa14-2), CD36 (HM36), CD64 (X54-5/7.1), CD206 (C068C2), CCR5 (HM-CCR5), PD-L1 (10F.9G2), CD80 (16-10A1), CD86 (GL-1) all from Biolegend, and PD-L2 (122) from eBioscience. Flow cytometry was performed using LSRII cytometer (BD Biosciences) and analyzed using Flowjo software (TreeStar). In some experiments, the stained C-MOs (CD115^+^Ly6G^−^CD11b^+^Ly6C^high^) in the blood or bone marrow, MAMPCs (F4/80^+^Ly6G^−^CD11b^high^Ly6C^high^), MAMs (F4/80^+^Ly6G^−^CD11b^high^Ly6C^low^), and RMACs (F4/80^+^Ly6G^−^CD11b^low^Ly6C^low^) in the lung of E0771-LG-injected mice were sorted using FACS Artia II (BD Biosciences).

### Microarray Analysis

We sorted C-MOs from the bone marrow and MAMPCs and MAMs from the lung with E0771-LG metastatic tumors (*N* = 3 per group) as described above. We extracted RNA from these cells by RNeasy Micro Kit (Qiagen) and used this for hybridization on Affymetrix MoGene 2.0 ST chip. Datasets were annotated and normalized using the robust multichip average algorithm (*rma)* from the GenePattern platform. All statistical calculations were performed in R programming language (version 3.2.3). The dataset was analyzed using the *oligo* package from Bioconductor. Multiple probes were collapsed to single gene using the average expression (*avereps* function). Using the *Limma* package, a linear model was fitted for the identification of differentially expressed genes. Genes with FDR ≤ 0.05 and log_2_FC ≥ +1.0 (upregulated) or log_2_FC ≤ −1.0 (downregulated) are considered to be differentially expressed. Venn diagrams were drawn using the differentially expressed genes between the populations. Heat maps were drawn using the *gplots* package on the differentially expressed genes between MAMPC and C-MO populations. A clustering method was set to complete and distance measure to Pearson correlation.

### Real-time Quantitative RT-PCR Analysis

We isolated total RNA from C-MOs, MAMPCs, MAMs, and RMACs as described above, and performed reverse transcription using oligo(dT)18 primers with SuperScript III (Invitrogen). Real-time quantitative PCR was performed using SYBR master mix (Invitrogen) on the Applied Biosystems 7500 Real-Time PCR System (Applied Biosystems) by the following program: preheating at 95°C for 10 min, 40 cycles of amplification consisting of 15 s denaturing at 95°C, 30 s annealing at 61°C, and 20 s extension at 72°C. Relative expression of target genes was determined according to ΔΔ*C_t_* with normalization to *Gapdh* expression. Primers used for PCR were: *Adm*, 5′-CACCCTGATGTTATTGGGTTCA-3′ and 5′-TTAGCGCCCACTTATTCCACT-3′; *Arg1*, 5′-CTCCAAGCCAAAGTCCTTAGAG-3′ and 5′-AGGAGCTGTCATTAGGGACATC-3′; *Cd163*, 5′-GGTGGACACAGAATGGTTCTTC-3′ and 5′-CCAGGAGCGTTAGTGACAGC-3′; *Hmox1*, 5′-AAGCCGAGAATGCTGAGTTCA-3′ and 5′-GCCGTGTAGATATGGTACAAGGA-3′; *Mrc1*, 5′-CTCTGTTCAGCTATTGGACGC-3′ and 5′-CGGAATTTCTGGGATTCAGCTTC-3′; *Stab1*, 5′-GGCAGACGGTACGGTCTAAAC-3′ and 5′-AGCGGCAGTCCAGAAGTATCT-3′; *Gapdh*, 5′-AGAACATCATCCCTGCATCC-3′ and 5′-CACATTGGGGGTAGGAACAC-3′.

### Blockade of CSF1 Receptor

For genetic depletion of CSF1 receptor, we gave doxycycline (SIGMA, 20 mg/mL) in drinking water with 5% w/v sucrose to the rtTA:tetO-Cre:*Csf1r*^F/F^ (*Csf1r* cKO) mice from 7 days after tumor injection to the endpoint. For pharmacological CSF1R inhibition, we gave BLZ945 (Selleckchem, 4 mg/20 g mouse, oral gavage) from 7 days after tumor injection to the endpoint.

### *In Vitro* CD8^+^ T Cell Cytotoxicity Assay

We isolated CD8^+^ T cells from the spleen of C57BL/6mice by EasySep Mouse CD8^+^ T Cell Isolation Kit (Stemcell technologies). 1 × 10^5^ of CD8^+^ T cells were cultured with or without 1 × 10^5^ of MAMPCs, MAMs, or RMACs from the E0771-LG tumor-bearing lung in enriched DMEM (20% v/v FBS, 2 mM l-glutamine, 1% v/v non-essential amino acid, 1 mM sodium pyruvate, 50 nM 2-mercaptoethanol, 100 U/mL penicillin, 100 µg/mL streptomycin) containing 80 U/mL IL-2, 2 µg/mL anti-CD3ε (145-2C11, Biolegend), and 5 µg/mL anti-CD28 (37.51, Biolegend). In some experiment, we added N^G^-Methyl-l-arginine acetate salt (L-NMMA, SIGMA, 500 µM), N^ω^-hydroxy-nor-arginine (nor-NOHA, Cambridge Bioscience, 500 µM), catalase (SIGMA, 1,000 U/mL), and superoxide dismutase (SOD, SIGMA, 200 U/mL) into the culture. After 4 days, CD8^+^ T cells (non-adherent) were collected and resuspended in enriched DMEM including 1,000 U/mL IL-2. The activated CD8^+^ T cells (effector) were then cocultured with 1 × 10^3^ of E0771-LG:NLR cells (target) at different effector/target ratio in the presence of fluorogenic caspase-3 substrate (NucView488, Biotium) in 96-well plates coated with basement membrane extract (Geltrex, Gibco). The cultured cells were imaged by IncuCyte Zoom Live-Cell Analysis System (Essen Bioscience) for 48 h, and number of apoptotic cancer cells (red/green double positive) were counted using IncuCyte S3 software.

### Statistical Analysis

Sample size was determined for power based on a relative SD from our previous studies. All samples were collected independently and analyzed by at least two independent experiments. Data were analyzed by Student’s *t*-test and are expressed as mean ± SEM. *P*-values <0.05 were considered significant.

## Results

### C-MO Differentiate into a Distinct Population That Gives Rise to MAMs in the Metastatic Site

**Figure 1:**
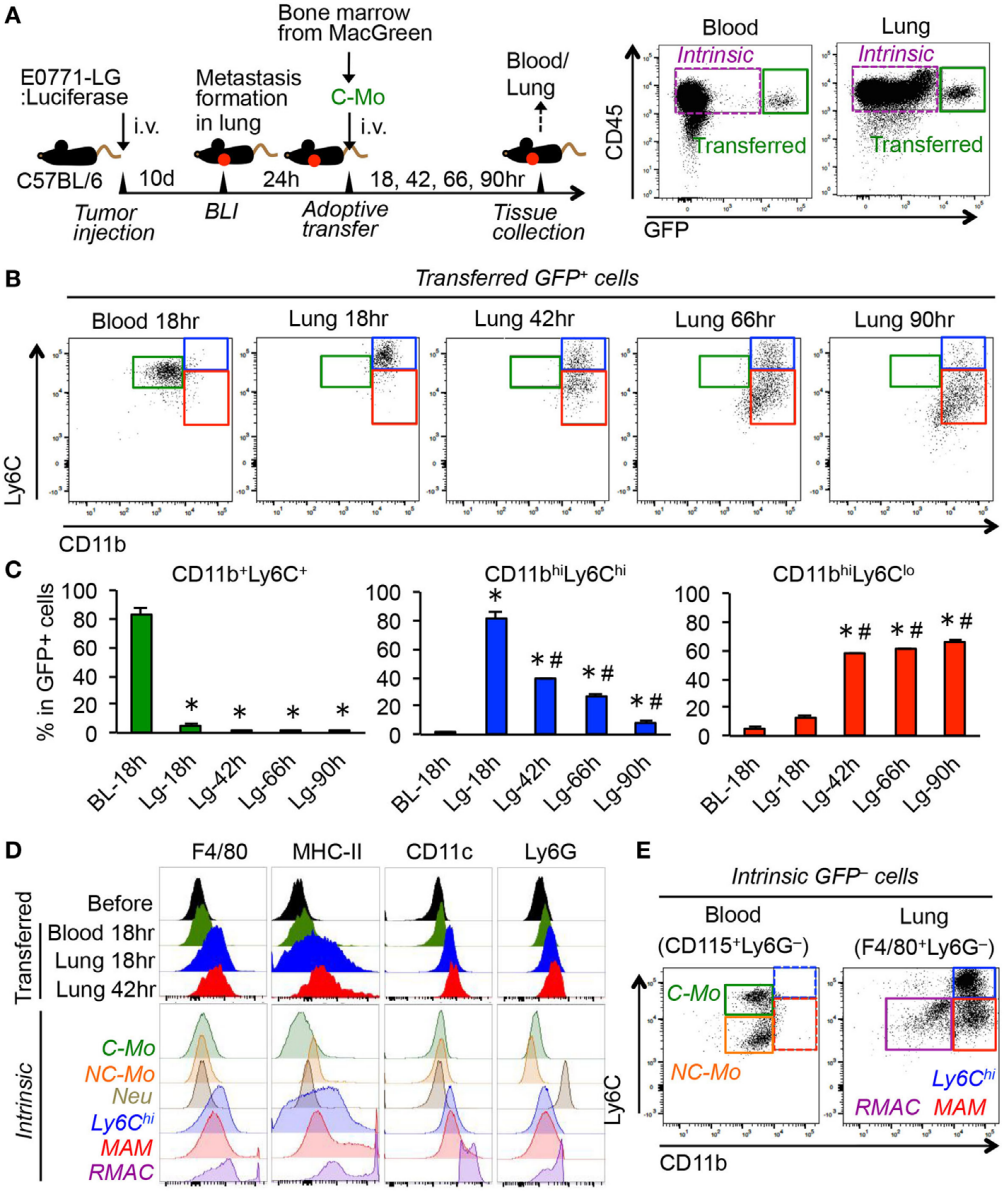
Adoptively transferred classical monocytes (C-MOs) differentiate into a distinct myeloid population that gives rise to metastasis-associated macrophages (MAMs) in the metastatic site. **(A)** A scheme of the monocyte tracking experiment (left) and representative dot plots showing the transferred (GFP^+^) and intrinsic (GFP^−^) cells in the blood and lung of the tumor-bearing monocyte-transferred mouse (right). **(B)** Representative dot plots showing expression of CD11b and Ly6C in the transferred GFP^+^ cells in the blood and lung of tumor-bearing mice after the indicated time posttransfer (*n* = 3/group, two independent experiments). Three typical populations characterized as CD11b^+^Ly6C^+^ (green), CD11b^high^Ly6C^high^ (blue), and CD11b^high^Ly6C^low^ (red) were shown. **(C)** Percentage is shown of CD11b^+^Ly6C^+^, CD11b^high^Ly6C^high^, and CD11b^high^Ly6C^low^ populations in GFP^+^ cells (*n* = 3/group, two independent experiments). Data are mean ± SEM, *P < 0.01 vs. blood 18 h (BL-18 h), ^#^*P* < 0.01 vs. lung 18 h (Lg-18 h). **(D)** Representative histograms showing expression of indicated markers in the transferred GFP^+^ cells in the blood and lung as well as GFP^+^ cells before transfer (before). As a control, expressions of markers in intrinsic cells from the tumor-bearing mouse are shown in the bottom. C-MO, non-classical monocytes (NC-MO), and neutrophils (Neu) were identified in blood, and CD11b^high^Ly6C^high^ cells (Ly6C^hi^), MAM, and resident macrophages (RMAC) were detected in the metastatic lung. Data are representative of two independent experiments with three mice per group. **(E)** Representative dot plots showing expression of CD11b and Ly6C in the intrinsic CD115^+^Ly6G^−^ cells in the blood and F4/80^+^Ly6G^−^ cells in the lung from the E0771-LG-injected C57BL/6 mice used in **(B)** (*n* = 3, two independent experiments). Three typical populations characterized as C-MO (green), NC-MO (orange), CD11b^high^Ly6C^high^ (blue), MAM (red), and RMAC (purple) were shown.

To understand the fate of C-MOs in the metastatic sites, we utilized MacGreen mice on a C57/BL6 background in which myeloid cells including monocytes and macrophages express green fluorescent protein (GFP) under the regulation of the CSF1R promoter (25). We transferred purified C-MOs from MacGreen mice into the C57BL/6 mice that have developed metastatic tumors in the lung following injection of E0771-LG mouse breast cancer cells that colonize the lung and form metastatic tumors. We tracked the fate of the GFP^+^ cells in the blood and lung at 18, 42, 66, and 90 h postinjection (Figure 1A). Since it was impractical to collect significant numbers of monocytes from peripheral blood, we isolated the C-MOs from the bone marrow that were characterized as CD11b^+^Ly6C^+^CD115^+^ but did not express markers for neutrophils (Ly6G) or hematopoietic stem/progenitor cells (c-Kit and Sca-1) (Figure S1 in Supplementary Material). By 18 h posttransfer, almost all GFP^+^ cells in the blood remained in a CD11b^+^Ly6C^+^ population that is characteristic for the C-MOs (Figures 1B,C). In contrast, GFP^+^ cells in the metastatic lung expressed higher levels of CD11b and Ly6C and a majority of them were found as a CD11b^high^Ly6C^high^ population distinct from the GFP^+^ cells and the intrinsic C-MOs in blood (Figures 1B,C). By 42 h after transfer, the GFP^+^ cells in the metastatic lung still expressed higher level of CD11b than C-MOs. However, more than half of cells had reduced Ly6C expression and thereby shifted to a CD11b^high^Ly6C^low^ population that resembles the phenotype of MAMs (10). The ratio of the CD11b^high^Ly6C^low^ population increased by 90 h after transfer, and that of CD11b^high^Ly6C^high^ population concomitantly reduced (Figures 1B,C), indicating that CD11b^high^Ly6C^high^ GFP^+^ cells are progenitors of MAMs. Interestingly around 15% of GFP^+^ cells in the lung after 90 h were outside the CD11b^high^Ly6C^low^ gate probably due to the reduction in their CD11b expression (Figure 1B). In our model, the transferred C-MOs in the blood became non-classical monocytes (NC-MOs) characterized as CD11b^+^Ly6C^low^ by 42 h posttransfer (Figure S2 in Supplementary Material) consistent with previous studies (28). However, these cells were distinct from the GFP^+^ cells in the lung that were characterized as CD11b^high^Ly6C^low^. The GFP^+^ cells in the metastatic lung expressed higher levels of F4/80 and major histocompatibility complex II (MHC-II) than GFP^+^ cells in the blood and intrinsic C-MOs (Figure 1D), suggesting their commitment to a macrophage lineage. Given their low expression of CD11c and Ly6G, these cells were distinct from RMACs, dendritic cells, or neutrophils (Figure 1D). As shown in Figure 1E, the CD11b^high^Ly6C^high^ population was also found in the intrinsic F4/80^+^ cells that accumulated in metastatic lungs, indicating that their presence is not an artifact of adoptive transfer. Although the CD11b^high^Ly6C^high^ cells might be considered as monocytes, their expression of CD11b, Ly6C, F4/80, and MHC-II clearly distinguish them from circulating C-MOs (Figures 1B,D,E). Collectively, these results indicate that circulating C-MOs differentiate to MAMPCs characterized as CD11b^high^Ly6C^high^ once they migrate to the metastatic site. Hereafter we call the CD11b^high^Ly6C^high^ cells MAMPCs.

### MAMPC Accumulation in Metastatic Sites Is Increased during Metastatic Tumor Outgrowth

**Figure 2:**
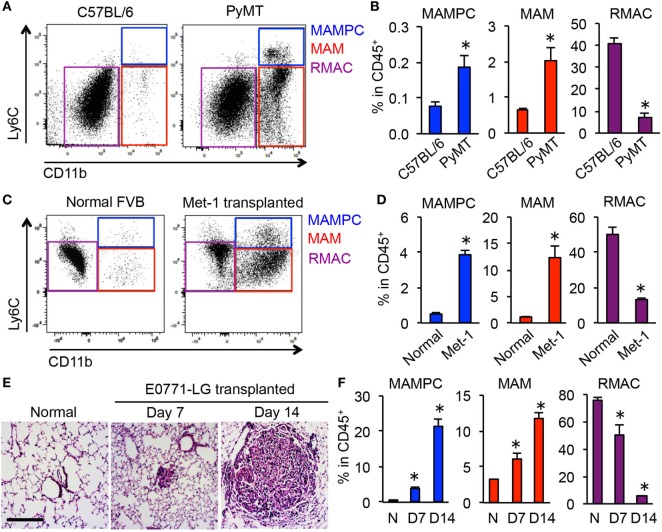
CD11b^high^Ly6C^high^ metastasis-associated macrophage (MAM) precursor cells (MAMPCs) accumulate in the lung during metastatic tumor growth. **(A)** Representative dot plots of the lung of normal C57BL/6 and BL6;PyMT mice that have established metastatic tumors (*n* = 3, three independent experiments). Cells were first gated as CD45^+^F4/80^+^, and then MAMPCs (blue), MAMs (red), and resident macrophages (RMACs) (purple) were defined as CD11b^high^Ly6C^high^, CD11b^high^Ly6C^low^, and CD11b^−^Ly6C^low^, respectively. **(B)** Relative numbers of MAMPCs, MAMs, and RMACs in the lung of C57BL/6 and metastasis-bearing PyMT mice (*n* = 3, three independent experiments). Data are mean ± SEM, *P < 0.05. **(C)** Representative dot plots of the lung from normal and metastasis-bearing FVB mice at 21 days after intravenous injection of Met-1 mouse mammary tumor cells (*n* = 3, two independent experiments). Cells were gated as described in **(A)**. **(D)** Relative numbers of MAMPCs, MAMs, and RMACs in the normal and Met-1 tumor-bearing (Met-1) lung at 21 days after tumor injection (*n* = 3, two independent experiments). Data are mean ± SEM, *P < 0.05. **(E)** Representative H&E-stained lung sections from normal C57BL/6 mice and from those transplanted with E0771-LG cells 7 or 14 days before isolation of the lung. Scale bar; 200 µm. **(F)** Relative numbers of cells with the phenotypes of MAMPCs, MAMs, and RMACs in the lung of normal (N, *n* = 4) and tumor-injected mice at days 7 and 14 post-E0771-LG injection mice (*n* = 3, two independent experiments). Data are mean ± SEM, *P < 0.05.

To investigate whether the MAMPCs accumulate in spontaneous pulmonary metastases, we analyzed lung metastatic lesions in PyMT mice on C57BL/6 background. Consistent with our results using transferred cells (Figure 1B), we found that F4/80^+^ cells in the spontaneous metastatic lung but not in the normal lung included a CD11b^high^Ly6C^high^ population (i.e., MAMPCs) that is distinct from MAMs (CD11b^high^Ly6C^low^) and RMACs (CD11b^low^Ly6C^low^) (Figure 2A). The relative numbers of MAMPCs as well as MAMs were significantly higher in the metastatic lung compared with the normal lung (Figure 2B), whereas the ratio of RMACs was relatively low due to the recruitment of myeloid cells to the tumors. We also analyzed another metastatic breast cancer model, i.e., FVB mice intravenously injected with Met-1 mouse mammary tumor cells (27). Consistent with the other two models, we found a significant increase in the numbers of MAMPCs and MAMs in the lung with metastatic tumors compared with normal lung (Figures 2C,D), suggesting that accumulation of the CD11b^high^Ly6C^high^ MAMPCs in the metastatic site is not a model-dependent artifact. To understand the timing of MAMPC accumulation in the metastatic lung, we utilized the experimental metastasis model using E0771-LG cells in which intravenously transplanted cancer cells reproducibly develop micro-metastases in the lung by day 7 that subsequently grow into macro-metastatic lesions by day 14 (Figure 2E). We found that the ratio of cells in the MAMPC gate increased by day 7 and further increased by day 14 (Figure 2F). Similarly, accumulation of cells in the MAM gate but not RMACs was increased when metastatic tumors grow in the lung (Figure 2F). Taken together, these results indicate that accumulation of MAMPCs is associated with the metastatic tumor outgrowth in mouse models of metastatic breast cancer.

### Morphology and Gene Profile of MAMPCs Are Distinct from C-MOs but Similar to MAMs

**Figure 3:**
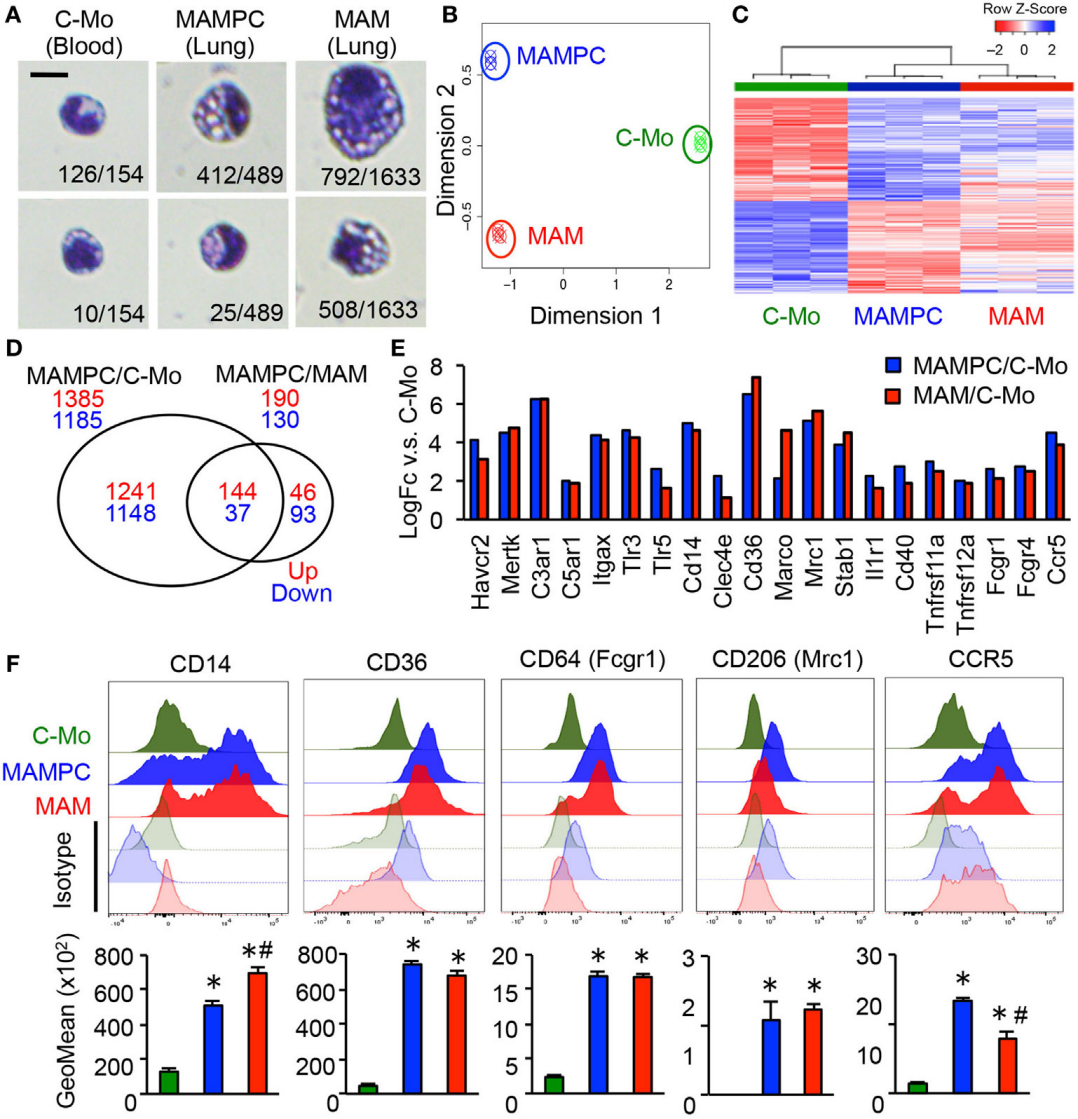
The metastasis-associated macrophage (MAM) precursor cells (MAMPCs) are morphologically and transcriptionally distinguishable from classical monocytes (C-MOs) and MAMs. **(A)** Representative morphology of C-MOs in the blood, and MAMPCs and MAMs in the lung of mice with E0771-LG metastatic tumors. Scale bar; 10 µm. The number of cells with the indicated morphology in the total counted cells is shown. **(B)** Unsupervised multidimensional scaling (MDS) plot of the normalized gene expression of RNA isolated from C-MOs, MAMPCs, and MAMs (*n* = 3/group). C-MOs were isolated from the bone marrow and MAMPCs and MAMs were isolated from the lung of mice bearing E0771-LG metastatic tumors. **(C)** Unsupervised hierarchical clustering of differentially expressed genes (FDR < 0.05) between MAMPC and C-MO populations. Columns indicate samples, rows indicate genes, and color intensity represents the *Z*-score-transformed RNA expression values. Samples are clustered using complete linkage and Pearson correlation. **(D)** Venn diagram of the commonly regulated genes in MAMPCs compared with C-MOs or MAMs (log_2_FC more or less than −1/1, FDR = < 0.05). **(E)** Genes encoding macrophage receptors that were upregulated in MAMPCs (blue) and MAMs (red) compared with IMs (logFC > 1, *P* < 0.01). Genes were clustered according to their ligands, i.e., phosphatidylserine receptors (*Havcr2, Mertk*), complement receptors (*C3ar1, C5ar1, ItgaX*), Toll-like receptors and coreceptor (*Tlr3, Tlr5, Cd14*), C-type lectin (*Clec4e*), scavenger receptors (*Cd36, Marco, Mrc1, Stab1*), cytokine and chemokine receptors (*Ilr1, Cd40, Tnfsf11a, Tnfsf12a, Ccr5*), and Fc receptors (*Fcgr1, Fcgr4*). Data on expression values are presented as mean ± SEM. Note that the scale is exponential. **(F)** Representative histogram (top) and mean fluorescent intensity (bottom) of indicated proteins in C-MOs, MAMPCs, and MAMs (*n* = 3, two independent experiments). Blood (for C-MOs) and lung digestion (for MAMPCs and MAMs) were prepared from E0771-LG-injected C57BL/6 mice at 14 days posttumor injection and stained with antibodies for indicated markers or isotype IgG. Data are mean ± SEM, *P < 0.01 vs. C-MO, ^#^*P* < 0.01 vs. MAMPC.

To further characterize MAMPCs, we analyzed the morphology of cells isolated by gating C-MOs, MAMPCs, and MAMs from the blood and lung of mice that have developed metastatic tumors by E0771-LG cells but have not received monocyte transfer. Most of the cells sorted by C-MO gate from the blood (126/154, 88%) showed the typical morphology of monocytes (Figure 3A), i.e., bi-lobate nucleus and a cytoplasm devoid of granules (29). In contrast, the majority of the cells sorted by MAMPC gate from the metastatic lung (412/489, 84%) were distinguishable from C-MOs by their larger cellular size (6.7 ± 0.05 and 5.7 ± 0.05 μm in diameter for MAMPCs and C-MOs, respectively, *P* < 0.01) and a vacuolated cytoplasm (Figure 3A), whereas a minority of them showed a similar morphology with C-MOs (25/489, 5%). Cells with cytoplasmic vacuoles were rarely found in the C-MO gate (10/154, 6%). In contrast, the majority of cells sorted by the MAM gate from the metastatic lung (1,300/1,633, 80%) had vacuoles in their cytoplasm. More than half of these vacuolated cells in MAM gate (792/1,300, 61%) were larger than MAMPCs (8.7 ± 0.05 μm and 6.7 ± 0.05 in diameter for MAMs and MAMPCs, respectively, *P* < 0.01), whereas 39% (508/1,300) of cells were comparable in size (6.2 ± 0.05 μm) with MAMPCs (Figure 3A). These results suggest that MAMPCs are in an intermediate stage of differentiation from C-MOs to MAMs.

To identify similarity and difference between C-MOs, MAMPCs, and MAMs we compared gene expression profiles of these cells by microarray analysis. To obtain sufficient cells, C-MOs were isolated from the bone marrow. Unsupervised multidimensional scaling plot of all expressed genes showed distinct clusters of the three populations, suggesting that MAMPCs are a distinct cell type from C-MOs and MAMs (Figure 3B). On the other hand, differential expression analysis and hierarchical clustering showed that there were fewer differentially expressed genes between MAMPCs and MAMs compared to C-MOs (Figures 3C,D; Table S1 in Supplementary Material), suggesting the phenotypic similarity between MAMPCs and MAMs. The array data also indicated that MAMPCs and MAMs both expressed higher levels of key macrophage receptor genes (30) than C-MOs, which included genes encoding phosphatidylserine receptors (*Havcr2, Mertk*), complement receptors (*C3ar1, C5ar1, ItgaX*), Toll-like receptors and coreceptor (*Tlr3, Tlr5, Cd14*), C-type lectin (*Clec4e*), scavenger receptors (*Cd36, Marco, Mrc1, Stab1*), cytokine and chemokine receptors (*Ilr1, Cd40, Tnfsf11a, Tnfsf12a, Ccr5*), and Fc receptors (*Fcgr1, Fcgr4*) (Figure 3E). To validate the array data, we determined expression of these receptors by flow cytometry in C-MOs from the blood and MAMPCs and MAMs from the lungs of mice with metastatic tumors. We confirmed that MAMPCs and MAMs in the metastatic lung expressed significantly higher levels of CD14, CD36, CD64 (Fcgr1), CD206 (Mrc1) and CCR5 proteins compared with circulating C-MOs in the tumor-bearing mice (Figure 3F). Expression of other receptors could not be analyzed due to the lack of reliable antibodies for flow cytometry. Taken together, these results indicate that MAMPCs are morphologically and phenotypically closer to MAMs rather C-MOs from which they derive.

### CSF1R Is Required for MAMPCs to Express TAM Signature Genes but Not to Accumulate in the Metastatic Site

**Figure 4:**
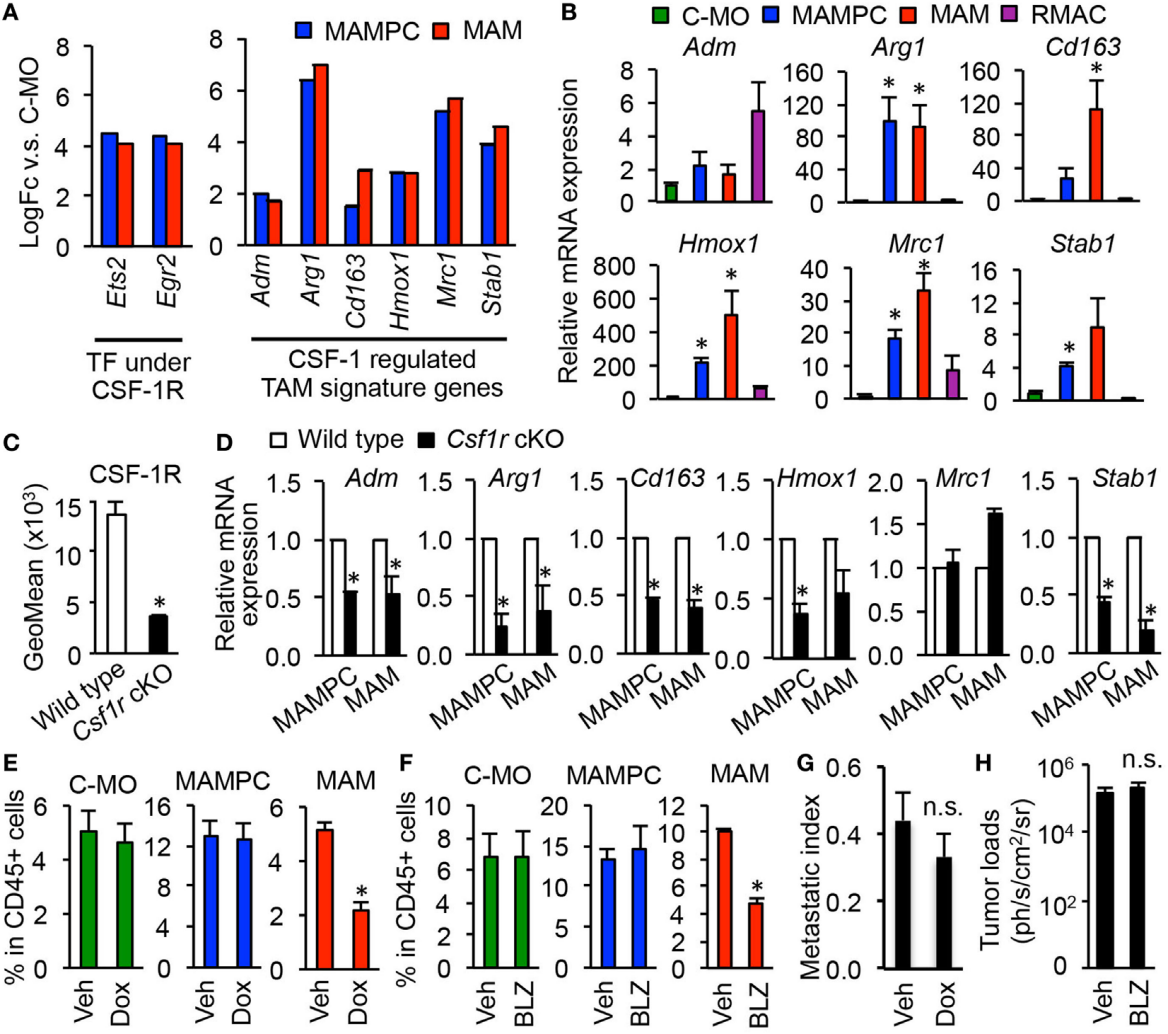
Colony-stimulating factor 1 (CSF1) signaling is required for tumor-associated macrophage (TAM) signature gene expression in metastasis-associated macrophage precursor cells (MAMPCs) but is not essential for their accumulation in the metastatic site. **(A)** Fold-change of genes encoding transcription factors (TFs) and TAM-signature genes (15) under CSF1 receptor (CSF1R) control determined by microarray analyses in Figure 3. Graphs show genes whose expression was higher in MAMPCs and MAMs than classical monocytes (C-MOs) (logFC > 1, FDR < 0.05). Data on expression values are presented as mean ± SEM. Note that the scale is exponential. **(B)** Relative mRNA expression assessed by quantitative RT-PCR in C-MOs (green), MAMPCs (blue), MAMs (red), and resident macrophages (RMACs) (Purple) (*n* = 3, two independent experiments). C-MOs were isolated from blood, and MAMPCs, MAMs, and RMACs were isolated from the metastatic lung of mice injected with E0771-LG cells. Data are mean ± SEM, *P < 0.05 vs. C-MO. **(C)** Mean fluorescence intensity of CSF1R in circulating C-MOs from tumor-bearing C57BL/6 (wild type) and CSF1R conditional knockout (*Csf1r* cKO) mice treated with doxycycline from day 7 to day 14 after intravenous injection of E0771-LG cells (*n* = 3, two independent experiments). Data are mean ± SEM, *P < 0.01. **(D)** Relative mRNA expression assessed by real time RT-PCR in MAMPCs and MAMs isolated from the metastatic lung of wild-type and *Csf1r* cKO mice that were treated as described above (*n* = 3, two independent experiments). Data are mean ± SEM, *P < 0.05. **(E)** Relative numbers of C-MOs in the blood, and MAMPCs and MAMs in the metastatic lung of *Csf1r* cKO mice treated with doxycycline (Dox) or vehicle (Veh) as described above (*n* = 3/group, two independent experiments). Data are mean ± SEM, *P < 0.01. **(F)** Relative numbers of C-MOs in the blood, and MAMPCs and MAMs in the metastatic lung of C57BL/6 mice treated with BLZ945, a selective CSF1R antagonist (BLZ) or Veh from day 7 to day 14 after intravenous injection of E0771-LG cells (*n* = 3/group, two independent experiments). Data are mean ± SEM, *P < 0.01. **(G)** Lung metastatic burden quantified as a metastasis index that is equal to total metastasis volume normalized by total lung volume. *Csf1r* cKO mice were treated with Dox or Veh from day 7 to day 14 after intravenous injection of E0771-LG cells (*n* = 6/group, two independent experiments). Data are mean ± SEM, *P < 0.01. **(H)** Lung metastatic burden quantified by bioluminescence imaging. C57BL/6 mice were treated with BLZ945 (BLZ) or Veh from day 7 to day 14 after intravenous injection of E0771-LG cells (*n* = 3/group, two independent experiments). Data are mean ± SEM, *P < 0.01.

We next investigated whether MAMPCs require CSF1R signal that is known to be essential for the recruitment, differentiation, and survival of TAMs (5, 31, 32). Our microarray data suggest that CSF1R signaling is active in MAMPCs and the cells express transcripts for CSF1R regulated genes at higher abundance than C-MOs, as mRNA levels of direct effectors of CSF1R signaling pathway (i.e., *Ets2* and *Egr2*) were fourfold higher in MAMPCs compared with C-MOs (Figure 4A). A recent study using a glioblastoma mouse model has identified ten signature genes whose expression in TAMs is regulated by CSF1R signaling, i.e., *Adm1, Arg1, Cd163, Cdh1, F13a1, Hmox1, Il1r2, Mrc1, Serpinb2, and Stab1* (15). We thus examined our gene list to determine whether these CSF1R dependent TAM signature genes were upregulated in MAMPCs, and found that levels of six genes (*Adm1, Arg1, Cd163, Cdh1, Hmox1, Il1r2, Mrc1, and Stab1*) were significantly higher (logFC > 1.5, *P* < 0.01) in MAMPCs than C-MOs (Figure 4A). Expression of these genes were also higher in MAMs than C-MOs. To confirm the array data, we compared mRNA levels of the six genes in C-MOs from the blood, MAMPCs, MAMs, and RMACs from the lung of mice with metastatic tumors. We found that expression of *Arg1, Hmox1, Mrc1, and Stab1* was significantly higher in MAMPCs than C-MOs and RMACs (Figure 4B). To investigate the involvement of CSF1R signaling in the expression of these genes, we utilized *Csf1r* conditional knockout (*Csf1r*-cKO) mice in which *Csf1r* gene is deleted by doxycycline treatment (21). The *Csf1r*-cKO and control C57BL/6 mice that have developed micro-metastases in the lung (Figure 2E) were treated with doxycycline for 1 week. These treatments significantly reduced CSF1R expression in C-MOs (the precursors of MAMPCs) in the *Csf1r*-cKO but not wild-type mice (Figure 4C). We found that almost all of the TAM signature genes except for *Mrc1* were downregulated in MAMPCs as well as MAMs by loss of CSF1R (Figure 4D), suggesting that CSF1R signaling is necessary for MAMPCs to express MAM signature genes when they are differentiated from circulating C-MOs.

We then asked whether the activation of CSF1R is required for the accumulation of MAMPCs in the metastatic site. As we expected, 1-week of treatment with doxycycline after micrometastasis formation significantly suppressed the accumulation of MAMs. However, the treatment did not reduce the number of MAMPCs or C-MOs (Figure 4E). Likewise, 1-week treatment of tumor-injected C57BL/6 mice with a specific CSF1R antagonist BLZ945 reduced the number of MAMs in the lung without affecting the number of MAMPCs and C-MOs (Figure 4F). These results suggest that CSF1 signal is required for MAMPCs to acquire MAM-like phenotype whereas other signals might be involved in the full development of MAMPCs from C-MOs and their subsequent survival. Since MAMPCs continuously produce MAMs, a single treatment with CSF1R antagonist might not be sufficient in time or degree of inhibition to prevent metastatic tumor outgrowth. Consistently, the short-term blockade of CSF1R after micrometastasis formation showed negligible effects on the metastatic tumor expansion (Figures 4G,H), although long-term genetic MAM depletion through loss of *Csf1* substantially inhibits metastasis (10).

### MAMPCs Suppress Cytotoxicity of CD8^+^ T Cells *In Vitro*

**Figure 5:**
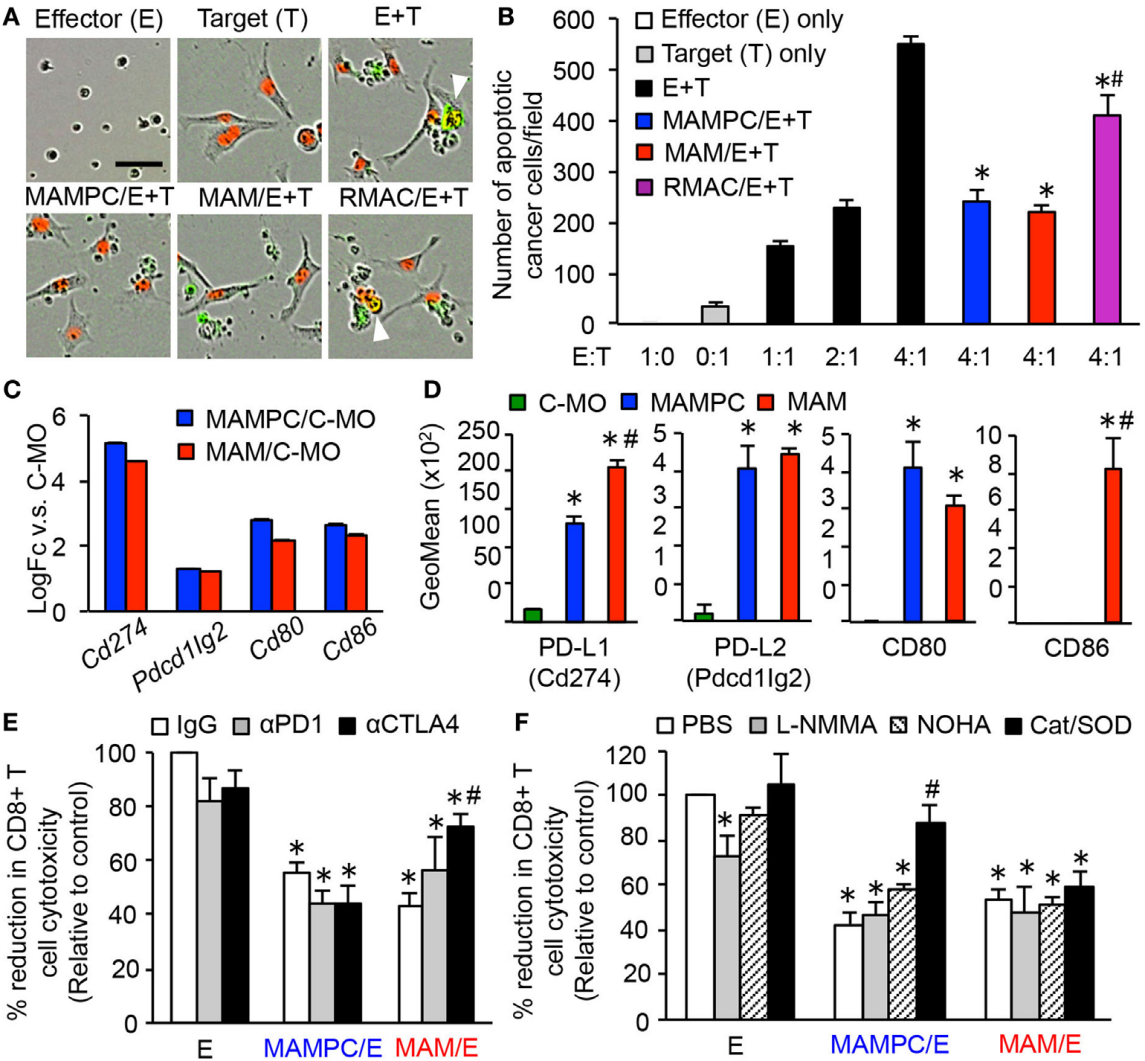
Metastasis-associated macrophage (MAM) precursor cells (MAMPCs) suppress cytotoxicity of CD8^+^ T cells through a reactive oxygen species (ROS)-mediated mechanism. **(A,B)** Effects of myeloid cells on the CD8^+^ T cell-induced tumor cell apoptosis. Splenic CD8^+^ T cells from normal C57BL/6 mice were cultured with anti-CD3/CD28 antibodies (effector; E) in the absence or presence of MAMPCs, MAMs, or resident macrophages (RMACs) from the metastatic lung of E0771-LG-injected mice (MAMPC/E, MAM/E, RMAC/E, respectively). The preincubated T cells were then isolated and cultured with E0771-LG cells expressing red fluorescent protein in the nuclei (target; T) at the indicated E:T ratio in the presence of green fluorogenic caspase-3 substrate. After 36 h, the number of apoptotic cancer cells indicated by red/green double positive nuclei was counted. **(A)** Representative images of cells cultured with the caspase-3 substrate for 36 h (E:T = 4:1). Scale bar; 50 µm, arrowhead; apoptotic cancer cell. **(B)** Number of apoptotic cancer cells cultured with preactivated CD8^+^ T cells (*n* = 3, two independent experiments). Data are mean ± SEM, *P < 0.01 vs. E + T (4:1), ^#^*P* < 0.01 vs. MAMPC/E + T. **(C)** Fold-change of genes encoding checkpoint T cell receptor ligands in MAMPCs and MAMs compared with classical monocytes (C-MOs) determined by microarray analyses in Figure 3 (logFC > 1, FDR < 0.05). Data on expression values are presented as mean ± SEM. Note that the scale is exponential. **(D)** Mean fluorescence intensity of checkpoint T cell receptor ligands assessed by flow cytometry in C-MOs, MAMPCs, and MAMs (*n* = 3, two independent experiments). Blood (for C-MOs) and lung digestion (for MAMPCs and MAMs) were prepared from E0771-LG-injected C57BL/6 mice at 14 days posttumor injection and stained with antibodies for indicated markers or isotype IgG. Data are mean ± SEM, *P < 0.01 vs. IM, ^#^*P* < 0.01 vs. MAMPC. **(E)** Effects of checkpoint inhibitors on the suppressive activity of MAMPCs and MAMs (*n* = 6, two independent experiments). CD8^+^ T cells were cultured with anti-CD3/CD28 antibodies and neutralizing antibodies for PD1 or CTLA4, or isotype IgG in the absence (effector; E) or presence of MAMPCs (MAMPC/E) or MAMs (MAM/E). Cytotoxicity of the precultured CD8^+^ T cells against E0771-LG cells at 4:1 E/T ratio were assessed as described above. Data are mean ± SEM that represent the ratio in number of apoptotic cancer cells relative to that induced by CD8^+^ T cells cultured with IgG in the absence of MAMPCs or MAMs (control). *P < 0.01 vs. control, ^#^*P* < 0.01 vs. IgG. **(F)** Effects of inhibitors of nitric oxide or ROS production on the suppressive activity of MAMPCs and MAMs (*n* = 6, two independent experiments). CD8^+^ T cells were cultured with anti-CD3/CD28 antibodies and L-NMMA, nor-NOHA, catalase and superoxide dismutase (SOD) (Cat/SOD), or vehicle (–) in the absence **(E)** or presence of MAMPCs (MAMPC/E) or MAMs (MAM/E). Cytotoxicity of the precultured CD8^+^ T cells against E0771-LG cells at 4:1 E/T ratio were assessed as described above. Data are mean ± SEM that represent the ratio of apoptotic cancer cells relative to that induced by CD8^+^ T cells cultured with PBS in the absence of MAMPCs or MAMs (control). *P < 0.01 vs. control, ^#^*P* < 0.01 vs. PBS.

The phenotype of MAMPCs with CD11b^high^Ly6C^high^Ly6G^−^ resembles that of a subpopulation of MDSCs called monocytic MDSCs (M-MDSCs) (33). Since MDSCs and TAMs have been reported to suppress antitumor immune reaction in primary tumor models (11, 34, 35), we hypothesized that MAMPCs and MAMs in the metastatic site also possess immune suppressive phenotypes and thus performed an *in vitro* CD8^+^ T cell cytotoxicity assay. In this assay, we cultured E0771-LG cancer cells expressing red fluorescent protein (target) with splenic CD8^+^ T cells that were preincubated with anti-CD3/CD28 antibodies (effector), and detected tumor cell apoptosis indicated by green fluorescence from a fluorogenic caspase-3 substrate under the microscopy (Figure 5A). As shown in Figure 5B, tumor cell apoptosis (i.e., number of red/green double positive cells) was induced by preactivated CD8^+^ T cells and enhanced in accordance with increased effector to target (E:T) ratios.

To test our hypothesis, we isolated MAMPCs, MAMs, and RMACs from the lung with metastatic E0771-LG tumors and cultured with splenic CD8^+^ T cells in the presence of anti-CD3/CD28 activating antibodies. We then isolated the CD8^+^ T cells and evaluated their cytotoxicity against E0771-LG cells. The CD8^+^ T cells cocultured with MAMPCs or MAMs showed significantly lower cytotoxicity compared with the cells without the coculture (Figures 5A,B). Although the CD8^+^ T cell cytotoxicity was also somewhat reduced by RMACs, their suppressive effect was significantly lower than MAMPCs or MAMs. Unfortunately we could not investigate suppressive effects of C-MOs due to the low number of cells collectible from the blood of tumor-bearing mice for this assay. Nevertheless and of importance, our data indicate that within the metastatic tissue MAMs and their immediate progenitors are immunosuppressive.

Our array data (Figure 5C) indicated that MAMPCs and MAMs compared with C-MOs expressed higher levels of mRNA coding PD-L1 (*Cd274*), PD-L2 (*Pdcd1lg2*), CD80, and CD86 that negatively regulate CD8^+^ T cell responses upon binding to checkpoint receptors, programmed cell death protein 1 (PD-1) and cytotoxic T-lymphocyte-associated protein 4 (CTLA4) (36). Flow cytometry confirmed that MAMPCs and MAMs expressed higher levels of PD-L1, PD-L2, and CD80 proteins compared with circulating C-MOs, whereas CD86 was expressed only MAMs (Figure 5D). We thus investigated their contributions to the CD8^+^ T cell suppression using antibodies against PD1 or CTLA4. Anti-CTLA4 blocking antibody partly but significantly increased the cytotoxicity of CD8^+^ T cell suppressed by MAMs whereas anti-PD1 antibody did not affect the suppressive activity of MAMs (Figure 5E), suggesting that CD80/CD86 expression on MAMs (Figure 5D) might have a role in the MAM-induced suppression. On the other hand, neither PD1 nor CTLA4 inhibition reversed the MAMPC-induced CD8^+^ T cell suppression (Figure 5E), suggesting that checkpoint receptors play a minor role in the suppressive mechanism of MAMPCs if any. We then investigated whether the MAMPC-induced suppression is mediated by arginase-1, inducible nitric oxide synthase (iNOS), and ROS that are key factors for MDSCs to exert immune suppressive activity (37). As shown in Figure 5F, combination of superoxide dismutase (SOD) and ROS-scavenging enzyme catalase significantly reversed the CD8^+^ T cell paralysis induced by MAMPCs, whereas this inhibition was not reversed by the iNOS inhibitor L-NMMA nor the arginase inhibitor NOHA. These results suggest that MAMPCs suppress CD8^+^ T cell cytotoxicity at least partly by a ROS-mediated but not by a nitric oxide (NO)-mediated mechanism. In contrast, these inhibitors had no influence in the MAM-mediated CD8^+^ T cell suppression (Figure 5F).

Taken together, this study identified a distinct population of MAM precursor in the metastatic sites that accumulate during the metastatic tumor growth by CSF1R independent mechanism, and suppress CD8^+^ T cell cytotoxicity by a ROS-mediated but checkpoint receptor-independent mechanism.

## Discussion

Myeloid cells such as macrophages, neutrophils, and MDSCs are known to accumulate in the tumor microenvironment and to actively promote the metastatic process (4). Using metastatic breast cancer models in mice, we have reported that MAMs, a distinct population of macrophages, abundantly accumulate in the tumor-challenged lung where they promote extravasation and survival of metastatic cancer cells (10). We have also identified that C-MOs preferentially migrate to the metastatic tumors and differentiate into MAMs (20). However, the fate and characteristics of the C-MOs after their migration into the metastatic site has not been elucidated.

Using a monocyte tracking method, we have identified here that circulating C-MOs (CD11b^+^Ly6C^+^) differentiate into a distinguishable myeloid cell population characterized by being CD11b^high^Ly6C^high^ immediately after migrating to the metastatic lung. Since the CD11b^high^Ly6C^high^ cells shift over time into a gate of MAMs (CD11b^high^Ly6C^low^) in the metastatic site, these cells can be identified as MAMPCs. Although C-MOs differentiate to CD11b^+^Ly6C^low^ (NC-MOs) in the blood, these cells were distinct from the CD11b^high^Ly6C^low^ MAMs by their lower expression of CD11b, F4/80, and MHC-II. Since C-MOs preferentially migrate to the metastatic lung than NC-MOs (20), the majority of MAMs in the lung are differentiated from C-MOs through MAMPCs, although our data does not exclude a minor contribution of NC-MOs recruited to the metastatic site or tissue-RMACs of embryonic origin as suggested by recent studies (38, 39).

Accumulation of the CD11b^high^Ly6C^high^ cells was found in the lung with metastatic mammary tumors developed by E0771-LG or Met-1 cells experimentally and by PyMT transgene spontaneously. Although the number of CD11b^high^Ly6C^high^ cells in the spontaneous model (PyMT) was lower than that in the experimental model (E0771), it is probably because PyMT mice on C57BL/6 background develop lower numbers and smaller tumors (0.3 ± 0.2 foci/mm^2^ lung, 0.3 ± 0.2 mm diameter/foci) compared with E0771-injected C57BL/6 mice (1.0 ± 0.3 foci/mm^2^ lung, 0.5 ± 0.1 mm diameter/foci, *P* < 0.01). Consistent with this interpretation, the number of the Ly6C^high^ cells in the experimental model correlated with tumor load in the lung. These results suggest that accumulation of the CD11b^high^Ly6C^high^ MAMPCs in the metastatic site is a common feature of breast cancer models in mice. Consistent with these data, a recent study has reported that CD11b^+^Ly6C^high^ cells are recruited to the lung with metastatic tumors developed by 4T1 mouse mammary tumor cells (40).

Metastasis-associated macrophage precursor cells are clearly distinguished from C-MOs or MAMs by their high CD11b and Ly6C expression as well as by their gene expression profile. Their low Ly6G expression excludes the possibility that they are neutrophils, as does their morphology. On the other hand, the phenotype of MAMPCs with CD11b^high^Ly6C^high^Ly6G^−^ resembles that of a subpopulation of MDSC called M-MDSCs (33). A recent report has proposed three criteria to identify cells as MDSCs; a population of cells (i) expanded compared with normal conditions, (ii) have typical phenotype of MDSCs (CD11b^+^Ly6C^hi^Ly6G^−^ for M-MDSC in mice) and (iii) possesses immune suppressive activity (33). We have shown that the MAMPCs are characterized as CD11b^+^Ly6C^hi^Ly6G^−^, expand in the lung with metastatic tumors compared with the normal lung, and suppress CD8^+^ T cell cytotoxicity *in vitro*. Therefore, the MAMPCs accumulate in the metastatic lung of breast cancer models can be identified as M-MDSCs.

Monocytic MDSCs have been defined as myeloid cells that are distinct from mature myeloid cells such as macrophages and neutrophils, but are morphologically and phenotypically similar to monocytes (34). In our model, the majority of MAMPCs in the metastatic lung had a larger cytoplasm containing vacuoles, which is clearly distinct from typical monocyte morphology but similar to MAMs, suggesting that M-MDSCs in the metastatic sites (i.e., MAMPCs) are committed to macrophage lineage compared with circulating C-MOs. Consistent with these data, the gene expression profile of MAMPCs was closer to that of MAMs than C-MOs, and the majority of MAMPCs expressed high levels of mature macrophage markers such as CD14, CD36, CD64, and CD206. Compared with C-MOs, MAMPCs expressed higher levels of TAM signature genes (i.e., *Arg1, Cdh1, Hmox1, Il1r2, Mrc1, and Stab1*) that were also highly expressed by MAMs, suggesting that differentiation of C-MOs to MAMPCs (M-MDSCs) is directed toward the tumor-promoting macrophages. Since circulating C-MOs expressed much lower levels of these macrophage markers compared with MAMPCs and only very minor population of C-MOs (6.5%) showed the MAMPC-like morphology with cytoplasmic vacuoles, differentiation of C-MOs to M-MDSCs occurs mainly in the metastatic sites. Since these cells accumulate in the metastatic tissue, this suggests that the rate of C-MO recruitment by high CCL2 (20) and their differentiation into MAMPCs exceeds the rate of MAMPC differentiation into MAMs, although the monocyte tracking experiment indicates that they will all do so eventually.

It has been reported that CD11b^+^Gr-1^+^ MDSCs transferred into the mice with C3 fibrosarcoma or EL4 lymphoma reduce their Gr1 (Ly6C/Ly6G) expression and express a macrophage marker F4/80 (41, 42). These results suggest that M-MDSCs can differentiate into TAMs in the tumor microenvironment, whereas the fate of M-MDSCs in the metastatic tumor microenvironment has not been fully identified. Our study shows direct evidence that the CD11b^high^Ly6C^high^ M-MDSCs originate from C-MOs and differentiate into MAMs (CD11b^high^Ly6C^low^) in the metastatic site. Consistent with our data, treatment of 4T1 tumor-bearing mice with all trans retinoic acid reduces the number of CD11b^+^Gr-1^+^ cells and concomitantly increases the number of CD11b^+^Gr-1^−^F4/80^+^ cells in the metastatic lung (43).

In this 4T1 tumor model, both Gr-1^+^ cells and F4/80^+^ cells from the metastatic lung have ability to suppress T cell proliferation and their cytokine secretion whereas the suppressive effects are stronger in F4/80^+^ cells than Gr-1^+^ cells, suggesting that MAMs are more potent immune suppressors than MDSCs (43). In contrast, our data indicate that CD11b^high^Ly6C^high^ cells (M-MDSCs/MAMPCs) and CD11b^high^Ly6C^low^ cells (MAMs) suppress cytotoxicity of preactivated CD8^+^ T cells at comparable levels. This discrepancy can be explained by heterogeneity of MDSCs in the former experiment, i.e., Gr-1^+^ cells include not only M-MDSCs (CD11b^+^Ly6C^high^Ly6G^−^) but also include less immune suppressive PMN-MDSCs (CD11b^+^Ly6C^low^Ly6G^+^) since anti-Gr-1 antibody recognizes both Ly6C and Ly6G (44). As anti-Ly6C antibody does not cross react with Ly6G, it is more specific to M-MDSCs. Consequently, our data suggest that both MAMs and their progenitor MAMPCs are potent immune suppressors in the metastatic tumors.

In our metastatic breast cancer model utilizing E0771-LG cells, suppression of CD8^+^ T cell cytotoxicity by MAMPC (M-MDSC) was reversed by a combination of catalase and SOD but not by L-NMMA, suggesting that M-MDSCs in the metastatic site of breast cancer use ROS rather than NO to suppress T cell functions. Consistent with these data, similar results were observed in 4T1 metastatic breast cancer model, i.e., T cell suppression by CD11b^+^Gr1^+^ MDSCs from metastatic lung was restored by catalase but not by L-NMMA (43). On the other hand, M-MDSCs isolated from the ascites or spleen of EL4 lymphoma-injected mice were reported to suppress T cell functions largely by production of NO and/or reactive nitrogen species (42, 44, 45). These results suggest that M-MDSCs might utilize different suppressive mechanisms in response to the tumor microenvironment that are determined by tumor types and location. Our results also suggest that MAMs and MAMPCs utilize different mechanism to suppress CD8^+^ T cell cytotoxicity, as inhibitors for ROS or NO production had no influence in the MAM-mediated CD8^+^ T cell suppression. Given the diversity and plasticity of suppressor myeloid cells, a combination of inhibitors might therefore be needed to block the immunosuppressive tumor microenvironment.

In a mouse model of pancreatic cancer, inhibition of CSF1R signaling suppresses TAMs accumulation and their immune suppressive functions, and thereby synergize with checkpoint-blockade immunotherapy (13). Given the effects of BLZ945 and anti-CTLA4 antibody on MAM functions in our model, the combination of CSF1R antagonist and checkpoint inhibitor may also be effective for metastatic breast cancer. On the other hand, our results suggest that MAMPCs, another immunosuppressive compartment in the metastatic site, might not be targeted by this strategy. Interestingly, it has suggested that accumulation of MDSCs requires two sets of signals that are largely mediated by tumor and tumor stromal cell-derived growth factors (e.g., GM-CSF, etc.) and proinflammatory cytokines (e.g., IL-6, etc.) (34). A recent study also shows that high mobility group box 1 (HMGB1) secreted from human breast cancer cells induces differentiation of monocytes into M-MDSCs and their survival (46, 47), suggesting that these cytokines will be potential therapeutic targets to prevent MAMPC accumulation and further improve immunotherapy effects metastatic breast cancer.

This study shows, to our knowledge for the first time, that MAMs are differentiated from a distinct population of precursor cells (MAMPCs) that phenotypically represent the cells originally described as M-MDSCs, and prevent T cell cytotoxicity at least *in vitro*. Characteristics of the MAMPCs identified in this study highlight the importance of further investigation of mechanisms behind their development in the metastatic site, which will lead to a novel and effective strategies to improve immunotherapy for metastatic breast cancer.

## Ethics Statement

All procedures involving mice were conducted in accordance with licensed permission under the UK Animal Scientific Procedures Act (1986) (Home Office license number PPL 70/8065).

## Author Contributions

TK and JP designed the research. TK and DB performed the experiments and analyzed the data. TK, DS, and NC established and performed the *in vitro* CD8^+^ T cell cytotoxicity assay. TK and YK generated the microarray data. LC and SF analyzed microarray data. TK, LC, and JP analyzed all the data and wrote the manuscript.

## Conflict of Interest Statement

The authors declare that the research was conducted in the absence of any commercial or financial relationships that could be construed as a potential conflict of interest.

## Abbreviations

C-MO, classical monocyte; NC-MOs, non-classical monocytes; TAM, tumor-associated macrophage; MDSC, myeloid-derived suppressor cell; MAM, metastasis-associated macrophage; MAMPC, MAM progenitor cell; RMAC, resident macrophage; CSF1R, colony-stimulating factor 1 receptor; PyMT, polyoma middle T oncoprotein; ROS, reactive oxygen species; SOD, superoxide dismutase.
