# Supplementary material for: Monocytes Differentiate to Immune Suppressive Precursors of Metastasis-Associated Macrophages in Mouse Models of Metastatic Breast Cancer
Source: Front Immunol. 2018 Jan 17;8:2004. doi: 10.3389/fimmu.2017.02004 (PMC5776392; doi:10.3389/fimmu.2017.02004)
Supplement: Supplementary file 1 [file Image_1.PDF]

Supplementary Material

Monocytes Differentiate to Immune Suppressive Precursors of Metastasis-Associated Macrophages

Takanori Kitamura*, Dahlia D. Shenton, Luca Cassetta, Stamatina Fragkogianni, Demi Brownlie, Yu Kato, Neil Carragher, Jeffrey W. Pollard

* **Correspondence:** Takanori Kitamura: tkitamur@exseed.ed.ac.uk

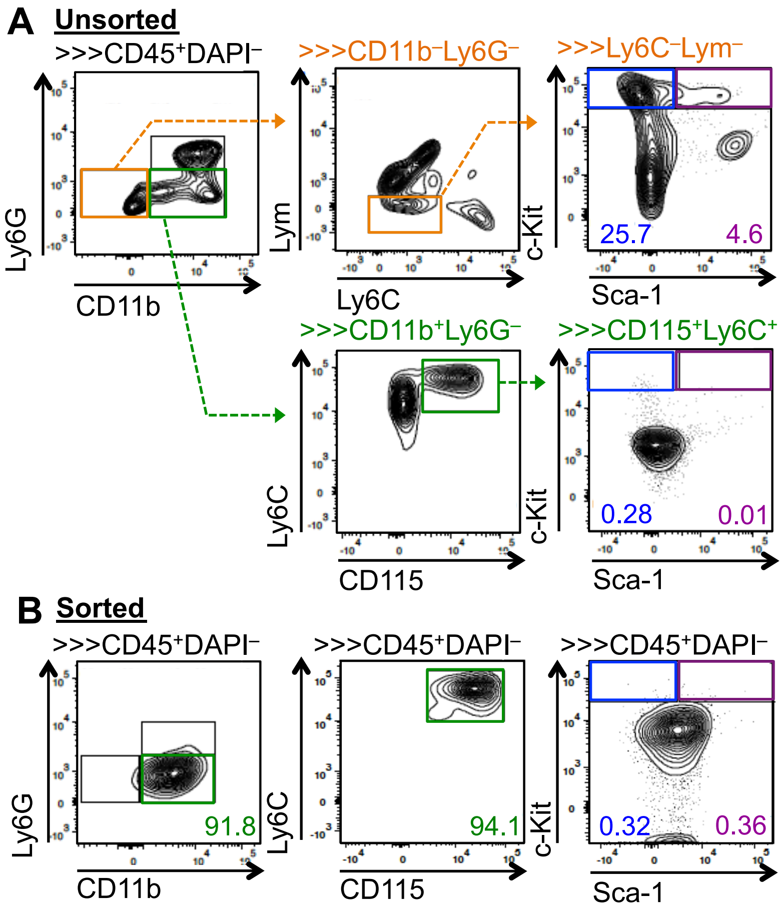

Supplementary Figure 1. Classical monocytes from the bone marrow are distinct from neutrophils or hematopoietic stem/progenitor cells. (A) Representative dot plots showing expression of c-Kit and Sca-1 in hematopoietic stem/progenitor cells (Top) and C-MOs (bottom) in the bone marrow of MacGreen mice. Cells that express low level of CD11b, Ly6G, Ly6C, and lymphoid markers (Lym) including CD3, B2.20, NK1.1, and TER119 were identified as lineage negative (Lin$^-$). Percentage of cells in the hematopoietic stem cell (Lin$^-$Sca-1$^+$c-Kit$^+$, purple) and progenitor cell (Lin$^-$Sca-1$^-$c-Kit$^+$, blue) are shown. **(B)** Representative dot plots showing expression of CD11b and Ly6G, CD115 and Ly6C, and c-Kit and Sca-1 in monocytes isolated from the bone marrow by magnetic sorting. Percentage of cells in the indicated gate are shown.
