# Supplementary material for: Monocytes Differentiate to Immune Suppressive Precursors of Metastasis-Associated Macrophages in Mouse Models of Metastatic Breast Cancer
Source: Front Immunol. 2018 Jan 17;8:2004. doi: 10.3389/fimmu.2017.02004 (PMC5776392; doi:10.3389/fimmu.2017.02004)
Supplement: Supplementary file 2 [file Image_2.PDF]

Supplementary Material

Monocytes Differentiate to Immune Suppressive Precursors of Metastasis-Associated Macrophages

Takanori Kitamura*, Dahlia D. Shenton, Luca Cassetta, Stamatina Fragkogianni, Demi Brownlie, Yu Kato, Neil Carragher, Jeffrey W. Pollard

*** Correspondence:** Takanori Kitamura: tkitamur@exseed.ed.ac.uk

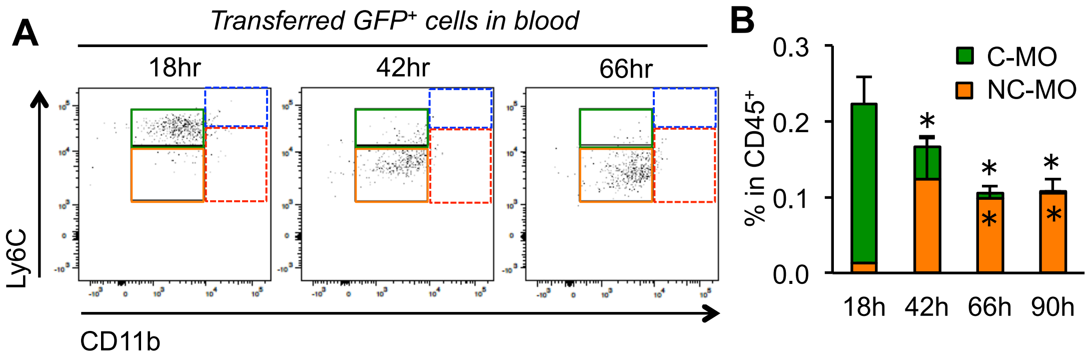

Supplementary Figure 2. Transferred classical monocytes differentiate into non-classical monocytes in the blood. (A) Representative dot plots showing expression of CD11b and Ly6C in the transferred GFP^+ cells in the blood of tumor-bearing mice after the indicated time after the IM transfer (n = 3/group, two independent experiments). Populations representing two monocyte subsets, i.e., classical monocyte (green) and non-classical monocyte (orange) were shown. The gates of two GFP^+ populations in the lung (Figure 1 A), i.e., $CD11b^{high}Ly6C^{high}$ (blue) and $CD11b^{high}Ly6C^{low}$ (red) were also shown. **(B)** Percentage is shown of transferred $CD11b^+Ly6C^+$ (classical monocyte, C-MO) and $CD11b^+Ly6C^-$ (non-classical monocyte, NC-MO) populations in $CD45^+$ cells (n = 3/group, two independent experiments). Data are means ± SEM, *P < 0.01 v.s. 18 hours.
